# A vision transformer architecture for the automated segmentation of retinal lesions in spectral domain optical coherence tomography images

**DOI:** 10.1038/s41598-023-27616-1

**Published:** 2023-01-10

**Authors:** Daniel Philippi, Kai Rothaus, Mauro Castelli

**Affiliations:** 1 NOVA Information Management School (NOVA IMS), Universidade Nova de Lisboa, 1070-312 Lisbon, Portugal; 2 Department of Ophthalmology, St. Franziskus Hospital, 48145 Muenster, Germany; 3 School of Economics and Business, University of Ljubljana, Ljubljana, Slovenia

**Keywords:** Computer science, Macular degeneration

## Abstract

Neovascular age-related macular degeneration (nAMD) is one of the major causes of irreversible blindness and is characterized by accumulations of different lesions inside the retina. AMD biomarkers enable experts to grade the AMD and could be used for therapy prognosis and individualized treatment decisions. In particular, intra-retinal fluid (IRF), sub-retinal fluid (SRF), and pigment epithelium detachment (PED) are prominent biomarkers for grading neovascular AMD. Spectral-domain optical coherence tomography (SD-OCT) revolutionized nAMD early diagnosis by providing cross-sectional images of the retina. Automatic segmentation and quantification of IRF, SRF, and PED in SD-OCT images can be extremely useful for clinical decision-making. Despite the excellent performance of convolutional neural network (CNN)-based methods, the task still presents some challenges due to relevant variations in the location, size, shape, and texture of the lesions. This work adopts a transformer-based method to automatically segment retinal lesion from SD-OCT images and qualitatively and quantitatively evaluate its performance against CNN-based methods. The method combines the efficient long-range feature extraction and aggregation capabilities of Vision Transformers with data-efficient training of CNNs. The proposed method was tested on a private dataset containing 3842 2-dimensional SD-OCT retina images, manually labeled by experts of the Franziskus Eye-Center, Muenster. While one of the competitors presents a better performance in terms of Dice score, the proposed method is significantly less computationally expensive. Thus, future research will focus on the proposed network’s architecture to increase its segmentation performance while maintaining its computational efficiency.

## Introduction

Age-related macular degeneration (AMD) is a disease of the human visual system that causes a progressive loss of central vision, leading to total blindness in its late stages. It is one of the major causes of severe vision loss in developed countries and mainly occurs in the population above 50 years of age^1^. AMD is a complex disease influenced by genetic, behavioral, environmental factors and their interactions^2^. In addition to a variety of influencing factors, AMD may have different manifestations^3^. One type, denoted as neovascular AMD, is characterized by abnormal vessel growth in the macular region, resulting in fluid leakages across various areas within, or underneath the retina^4^. Early stages of AMD show the presence of drusen and abnormalities in the retinal pigment epithelium (RPE). Later stages can be further subdivided into neovascular AMD and dry AMD^5^. Neovascular AMD (nAMD) is characterized by the presence of various lesions that occur in addition to the signs of the early stages. Primarily, these include intra-retinal fluid (IRF) and sub-retinal fluid (SRF), as well as the detachment of the RPE from the Bruch’s membrane (BM), called pigment epithelium detachment (PED)^5^. In 2022, the international AMD research group published a consensus AMD nomenclature^3^. In that work, several AMD biomarkers in optical coherence tomography images are outlined, including different kinds of fluids (intraretinal, subretinal, sub-RPE), different kinds of pigment epithelium detachment (serous, fibrovascular, drusenoid), drusen, subretinal drusenoid deposits (pseudo-drusen), types of retinal atrophies (complete or incomplete, with or without RPE-defect), SHRM (subretinal hyperreflective material), HRF (hyperreflective foci), fibrosis, RIP (rip of the retinal pigment epithelium). It turned out that for our investigated prognostic targets, IRF, SRF, and PED are the biomarkers with the highest relevance. For this reason, in this paper, we investigate the segmentation performance of our proposed approach considering the three features (IRF, SRF, and PED) with the highest impact on prognostic targets. IRF, SRF, and PED are also considered as the most relevant biomarkers for managing the treatment of nAMD^6^. The main type of treatment is the so-called antivascular endothelial growth factor (anti-VEGF), which is injected into the vitreous. Progress and response of the treatment are monitored by an imaging technique called optical coherence tomography (OCT), which allows for the visualization and quantification of biomarkers in the macula (= central retinal region)^1,4,7^. OCT is a non-invasive imaging technique that produces cross-sectional images of the retina^7^ and is state-of-the-art in the diagnosis and monitoring of nAMD^6^. A light beam traverses the frontal plane centered around the point of interest, usually the fovea centralis (foveola) or the papillary. At every position, it generates a depth profile, called amplitude scan (A-scan), by analyzing the spectrum of the back-reflection of the beam^8^. There exist various scan patterns (e.g. 3d-scan, radial scan, and raster scan) that specify the movement pattern of the light beam across the frontal plane^7^. In this work, we use the 3d-scan pattern, which gives the most holistic view of the retina and we focus on the area around the foveola as the point of interest. To initiate, continue, stop, or change the treatment of nAMD, the identification of presence and changes concerning IRF, SRF, and PED is crucial^7^. Automated systems to segment and quantify those lesions can be extremely useful to support clinical decision-making for an early diagnosis and a quantifiable treatment of nAMD. Ultimately, this can lead up to a patient individual predictions of treatment intensity.

However, the huge variations in the location, size, and texture of those lesions, as well as the presence of speckle noise in the images, make accurate segmentation a challenging task, which thus remains an active field of research. In OCT images, speckle is both the source of noise and the carrier of information. Arguably, without speckle, an OCT image visualizing dense tissue would be empty. However, speckle noise poses a considerable challenge to the segmentation of an OCT image, as it results in reduced contrast and unclear boundaries within the image^9^. Recent years have witnessed the implementation and adoption of deep learning^10^ (DL)-based systems in the domain of retinal lesion segmentation in OCT images. In particular, DL-based methods^11–13^ repeatedly outperformed conventional methods. They are unanimously based on convolutional neural networks (CNNs), deep architecture specifically designed for feature extraction in grid-like data, including image data. Those methods include multi-stage approaches consisting of a pre-processing stage followed by the actual segmentation network and an optional post-processing stage. In the pre-processing stage, the localization prior to the region of interest (ROI) is passed to the network by limiting the image boundaries to the retinal region. The application of post-processing helps to reject falsely segmented lesions by training an additional classifier or to combine lesion instances falsely interrupted by the background class. However, with the design of increasingly advanced architectures^14–16^, recent single-stage networks can learn the information induced through pre- or post-processing by themselves. This comes with the advantage of reduced computational complexity and, consequently, making these methods more clinically applicable^17^. Despite the advances provided by DL, there is still a need for more accurate automatic segmentation tools. To answer this call, our objective is to improve CNN-based methods’ performance in modeling long-range contextual information and reduce speckle noise to cope with domain-specific challenges. To achieve this objective, we rely on Vision Transformers (ViTs)^18^. ViTs have a larger receptive field and perform learned feature aggregation. Thus, they have a stronger localization ability and better propagation of contextual and semantic information through the network compared to CNN-based networks^19^. These advantages come at the cost of a higher demand for training data and higher computational costs. For this reason, amongst many recent publications, we rely on Swin-UNETR^20^ which combines all the advantages of ViTs, while compensating for their weaknesses. It uses a Swin Transformer^21^ as the backbone architecture to reduce computational complexity and combines it with a CNN-based decoder to reduce the hunger for training data.

In this work, we evaluate ViTs on the segmentation of retinal lesions in OCTs.

This work belongs to a recent research strand in which transformer-based architectures^22^ were considered for the analysis of OCT images. However, when compared to the existing contributions, several differences emerged. Playout and coauthors^23^ thoroughly investigated the performance of several transformers and compared it to traditional CNN-based models for retinal disease classification. Experimental results showed that Vision Transformers achieve comparable performance to CNNs in retinal disease classification and scale better with larger training sets than CNNs. Different from our study, Playout and coauthors did not consider segmentation tasks. However, the idea of adding adjustable stride and focused attention looks interesting, as it addresses the issue of efficiently using finer-grained image patches to increase performance. The shifted window mechanism considered in our approach addresses the same issue but looks more suited to be integrated into a segmentation network due to its cascading structure. Another recent contribution was proposed by Wang et al.^24^, who developed an architecture for the automatic segmentation of retinal edema lesions in OCT images. Thus, the authors focused on a different disease than the AMD we examined. Moreover, their manuscript differs in at least two aspects from our study: (1) concerning the encoding of long-range, multi-scale features, it used a CNN-based backbone and added a transformer at the bottleneck. On the other hand, in our study, we continuously extract global information throughout the encoder levels relying on the transformer’s self-attention mechanism. (2) A CNN-based feature extractor produces a semantic gap that is subsequently bridged by adaptively fusing multi-scale features, while our study assumes a weaker semantic gap thanks to a transformer-based feature extractor. Other relevant studies are the ones of Kihara^25^ and Jiang^26^, where transformers have been considered for analyzing OCT images. Kihara and coauthors focused on nonexudative macular neovascularization and proposed a transformer-based encoder-decoder architecture for the segmentation task. Similarly, Jiang and coauthors proposed a transformer-based model to classify OCT images of AMD and Diabetic Macular Edema (DME). In our study, we rely on a hybrid Transformer-CNN that, unlike the pure transformer used by Kihara, is less training data demanding and thus does not require pretraining. In particular, we evaluate the use of a shifted window transformer backbone which, contrary to the transformer backbones in the aforementioned studies^25,26^, can efficiently process more fine-grained image patches through the use of local self-attention. This follows the concept exploited in the work of Playout^23^, which increased the number of patches drawn from the image through convolutions with adjustable strides to improve classification performance and used focused attention to compensate for the additional memory requirements. Other contributions (like the work of Lee and coauthors^27^) concerning the use of less recent deep learning methods for AMD diagnosis and OCT image analysis have been proposed in recent years. However, considering that they are based on traditional CNNs architectures, we did not discuss their differences to our transformer-based approach. The key contributions of this study include **C.1**—the adaptation of Swin-UNETR to the purpose of automated segmentation of retinal lesions in nAMD SD-OCT scans; **C.2**—the quantitative and qualitative validation of the proposed method on unseen testing data and comparison with other state-of-the-art methods; **C.3**—the investigation of the effect of lesion size on the segmentation performance.

## Technical background

Recent years have witnessed the development of new deep architectures enriching CNNs with attention mechanisms^28–31^. Mathematically, attention is the process of mapping a query and a set of key-value pairs to an output. A compatibility function is used to calculate a set of weights based on the query and the corresponding key. The weights are used to compute the output as the weighted sum of the values^22^. Attention is commonly used in computer vision tasks to adaptively aggregate features that capture long-range contextual information^28^ and to suppress irrelevant parts of features while highlighting the relevant ones for a given task^32^. Attention can be divided into trainable and non-trainable, and the former can be further divided into hard and soft-attention^28^. In this work, we will focus on soft attention, as it is differentiable and thus can be trained in an end-to-end network architecture. The learned parameters come from three individual linear transformations, where sets of weights are learned to map the input into key, value, and query, respectively^22^. Soft attention can be further characterized depending on the size of the neighborhood (local or global)^31^, the type of compatibility function used to compute the weights (additive or multiplicative)^31^, and the input source (self, encoder-decoder)^22^.

The transformer architecture^22^ is the first model relying entirely on self-attention to compute representations of its input and output without using recurrence or convolutions. The original transformer architecture follows an encoder-decoder structure. In more detail, the encoder maps an input sequence to a sequence of continuous representations, which is then fed into a decoder. The decoder receives the output of the encoder together with the decoder output at the previous time step and generates an output sequence. This architecture was first developed for addressing natural language processing tasks^33^. Subsequently, Dosovitskiy^34^ proposed the first transformer for vision tasks, dubbed ViT. ViT was obtained by adapting the original transformer architecture to move from text processing to image processing. First, to simulate a sequence of words as input, an image of size H $\times$ W $\times$ C is reshaped into a sequence of flattened, non-overlapping 2d patches of size N $\times$ (P $\times$ P $\times$ C), where P is the pixel size of each patch, C is the number of input channels, and N the number of patches. Second, the fixed positional encoding was replaced with a learnable 1d encoding^35^.

The application of attention mechanisms in image segmentation improves the representational performance of the network. In particular, it results in a richer feature space, capturing long-range dependencies. The network learns to focus on relevant spatial regions (the “where”) or channels (the “what”) within a certain context^31^. Contrary to encoder-decoder attention mechanisms, self-attention is based solely on the given input feature maps and aggregates contextual information across the different dimensions of this input.

### From ViT to Swin transformer

The original ViT heavily relies on global self-attention for feature extraction with very little image-specific bias induced a priori. To improve its performance, Liu and coauthors proposed Swin Transformer^21^. It is designed as a general-purpose backbone for dense prediction tasks like object detection and image segmentation. The main observation is that for dense prediction tasks, translational invariance and the ability to process finer-grained patches are more important than instant global attention at every layer. Compared to the original ViT architecture, the Swin Transformer varies in three main aspects, namely the application of local self-attention, hierarchical feature maps, and relative position bias. In more detail, in local self-attention, heads (i.e., attention modules) of each layer only attend to a restricted non-overlapping group of patches, the attention windows. This reduces the computational complexity from quadratic to linear to the image size over global self-attention. Consequently, it allows for processing more fine-grained patches at comparable computational costs, which is important for dense prediction tasks like image segmentation. Hierarchical feature maps are used to create multi-scale outputs during feature extraction. After a user-defined number of consecutive transformer blocks with a constant patch resolution, the patch merging operation is performed at the start of a new layer. In patch merging, features related to a group of neighboring patches are concatenated, and a linear layer allows obtaining the vectorized embedding. This way, feature resolution is gradually decreased with deeper encoder levels, allowing for an in-place replacement of CNN-based backbones like ResNet and, thus, a smoother integration with decoders in U-Net-like architectures. Finally, instead of absolute positional embeddings, Swin Transformers learn a relative position bias inside the self-attention mechanism, achieving significant performance improvements. Here, an additional set of parameters is added to the computation of the attention coefficients, which learns the relative distance of each patch to every other patch.

## Materials and methods

### Dataset

In this work, we use a private dataset developed in collaboration with Franziskus Eye-Center in Muenster, Germany. All procedures performed in this study involving human participants were in accordance with the ethical standards of the institutional and/or national research committee and with the 1964 Helsinki Declaration and its later amendments or comparable ethical standards. Approval for the study was obtained from the University of Muenster Ethical Committee. Being a retrospective study, the Muenster Ethical Committee waived the need for informed consent. A cohort of nAMD patients treated with anti-VEGF therapy was selected. Out of those patients, we only consider OCT volumes that do not date back more than 18 months from the start of the treatment. Furthermore, we subsampled the OCTs to obtain an even distribution across these 18 months. All the images were acquired using a Spectralis SD-OCT from Heidelberg Engineering. Each volume contains 49 B-scans (i.e., cross-sectional images obtained from the combination of multiple A-scans) with a spatial resolution of 512 $\times$ 496. The training labels were generated through a manual annotation process performed by a group of professional ophthalmologists. We have defined a set of 17 AMD biomarkers and three retinal slab limits. We randomly selected 3842 B-scans (from 1400 randomly selected SD-OCTs) within the central 3 mm around the fovea. Four junior and one senior annotator annotated the B-scans. All graders have clinical ophthalmological experience and have undergone certification, with the annotation process documented in a detailed manual. The annotation was performed in a reading center environment using the COCO annotator^36^. Their work was supervised by a senior annotator who also performed a quality assessment on a certification dataset containing 50 slices that were selected to capture a broad spectrum of cases. To make the time-consuming process of manual annotation as efficient as possible, we focus on slices that cut through the macula. This is the region where IRF, SRF, and PED have the highest clinical relevance, and it should roughly be positioned around the center slice. Therefore, we randomly select five B-scans (slices) of every volume, using a gaussian probability distribution centered around the middle slice. In terms of preprocessing, to define the ROI, we used the internal limiting membrane (ILM) as the upper limit, which was segmented by the OCT machine and extracted from the raw OCT file. In particular, the area in the depth dimension of the retinal region, where lesions can occur, is defined between the ILM and the BM^4,7^. The BM was pre-segmented using polynomial regression. Inspired by the work of Russakoff^37^, we considered a fixed offset of 390$\mu$m below the BM as the lower limit of the ROI to capture choroidal information of a fixed area below the BM. The pixel values outside the ROI were replaced with zero. To improve the image quality, we applied contrast-limited adaptive histogram equalization (CLAHE)^38^. Here, an image is equally divided by a horizontal and a vertical factor ($grid\_size$) into non-overlapping regions. For each region, a histogram is calculated and redistributed to not exceed a $clip\_limit$ value. Based on the resulting contrast-limited histograms, a grayscale mapping is determined^39^. Finally, each pixel of the image is mapped to the grayscale mappings of the four nearest regions. In this work, we used the implementation by OpenCV 4 (https://opencv.org/), with a $grid\_size$ of 8 $\times$ 8 and a $clip\_limit$ of 1.

Furthermore, we applied a horizontal and vertical registration of the images to reduce the variance of the training data. We took the left and rightmost points of the RPE layer and used shearing so that both RPE endpoints lay on the same horizontal axis. We subsequently translated the image in the vertical direction, so that the RPE endpoints are positioned at 65% from the top of the image. Concerning training, validation, and testing, we only consider B-scans that contain at least one type of lesion (for a total of 3842 B-scans). Table 1 reports the distribution over the classes. Furthermore, each B-scan is resized to a dimension of 224 $\times$ 224 pixel. To add some regularization, we augment the training data by rotating the image by ± 20 degrees at a probability of 50%.

**Table 1:** Number of B-scans per class.

Class	Absolute	Relative
PED	3203	0.84
IRF	1783	0.47
SRF	1416	0.37

The relative values are based on the total number of B-scans (3842).

### Evaluation metrics

We consider the problem at hand as a multi-class, single-label task. The four individual classes are background, IRF, SRF, and PED. However, when generating the labels, the class masks are overlayed, resulting in a single label passed to the network, with pixel values chosen from the set of 0 = BG, 1 = IRF, 2 = SRF, and 3 = PED respectively. Thus, a co-occurrence of multiple classes at a particular pixel is excluded. We argue that the information loss due to overlaying the class masks is very limited since different types of lesions generally do not occur in the same position. In the selection and calculation of the metrics, we follow the guidelines^40^ of evaluation metrics for medical image segmentation: We use the Dice score^41^ as the main metric for performance evaluation. Additionally, we report sensitivity and specificity for method comparability, both individually per class and as a weighted aggregation. We use the image-wise averaging to aggregate the metrics to allow for an even contribution of all images and to account for the class imbalance. Here, the metrics are calculated for each image separately, and per-class metrics are averaged over the dataset. To obtain a final metric, we average the scores over the classes, weighted by the class distribution of the dataset. When computing the scores, we ignore images where either reference or prediction masks for a class are empty to avoid the zero division issue^42^. Furthermore, we will provide boxplots depicting the distribution of the metrics over the dataset, as well as sample visualizations of references and predictions for the considered methods.

#### Metric comparison and interpretation

Low-density predictions show numerous tiny holes inside the segmented area, while the area of the reference is solid. If density and the general shape and location are more important than the precise contours of an object, overlap-based metrics, like Dice, are recommended, as, unlike distance-based metrics, they penalize low density^41^. In this work, the segmentation task is primarily aimed at a precise quantification of the segments’ volume. Consequently, we will use the Dice score as our main metric.

### The proposed framework

In this work, we use as segmentation network Swin UNEt TRansformers (Swin-UNETR)^20^, which is specifically designed for the task of medical image segmentation. It is an encoder-decoder-based Transformer-CNN hybrid, with a transformer-based encoder, skip connections, and a CNN-based decoder. Though the original paper^20^ described a 3d segmentation problem, we adapt the following description to the 2d task to align with the problem at hand. The encoder uses a Swin Transformer backbone. It consists of five decoder stages and four encoder stages with a total of 12 layers ([2, 2, 6, 2] per stage). At each stage, patch resolution is increased, starting at 2 $\times$ 2 pixels per patch and reaching 32 $\times$ 32 pixels. The model’s feature (i.e., embedding) size is set to 24 and is doubled at every encoder stage. The remaining network repeatedly makes use of a residual block composed of two 3 $\times$ 3 convolutions followed by an instance normalization. At each encoder stage, the output is reshaped into the spatial dimensions and run through a residual block to be concatenated with the output of the previous decoder stage. The result of the concatenation is then run through another residual block, followed by a transposed convolution layer, that increases the resolution of the feature maps by factor 2, to conclude the current decoder stage. The resulting network has a decoder depth of 5 and a total of 6.3 Mio. parameters.

### Considered CNNs

We compared the performance achieved by the considered Swin-UNETR against state-of-the-art CNNs specifically developed for medical image segmentation.

#### U-Net

The U-Net^43^ is a commonly used, CNN-based, encoder-decoder architecture designed for medical image segmentation. It consists of a contracting path (encoder) and a symmetric expansive path (decoder). The encoder consists of cascaded convolutional blocks, ReLU activations, and a max-pooling operation. It generates low-level but high-resolution features in the early layers and increasingly higher-level semantic features in the deeper layers. The decoder consists of transposed convolutions, an un-pooling layer, and ReLU activations symmetric to the ones in the encoder. The decoder is built to aggregate multi-level features and capture multi-scale context information. It continuously increases the spatial resolution of the feature maps to regain the original input’s resolution. Furthermore, skip connections are used to concatenate features from the decoder with the ones of the corresponding encoder level. Finally, the last layer is a 1 $\times$ 1 convolution, which maps the features of the previous layer to the desired number of classes^30^. Despite its success, the original U-Net fails to fully recover the spatial information lost during down-sampling and flawlessly to bridge the semantic gap between low and high-level features. Thus, many adaptations like spatial pyramid pooling (SPP), atrous spatial pyramid pooling (ASPP), or input pyramids have been proposed to improve segmentation accuracy^44^.

#### U-Net3+

U-Net3+^45^ builds on top of the original U-Net and the U-Net++^46^ to propose a position and boundary-aware network for medical image segmentation. The primary change is the redesigned, full-scale, skip connection mechanism to improve multi-scale feature aggregation and the deep supervision that learns hierarchical representations. Low-level, high-resolution features are important for a precise segmentation of boundary regions, while high-level features embody positional information needed to locate the lesions. Multi-scale feature extraction and aggregation is a widespread solution to merge these features. Full-scale skip connections consider features of all scales at each particular decoder stage, to improve multi-scale feature fusion. At one particular decoder, like in the vanilla U-Net, the feature maps of the corresponding encoder are passed directly. Additionally, feature maps of earlier encoder stages are passed through max pooling operations of increasing scale to match the spatial resolution of the current stage. Likewise, coarser feature maps are taken from the previous decoder layers and are up-sampled through bilinear interpolation. To unify the depth across the feature maps and reduce redundant information, feature maps of every scale are run through individual 3 $\times$ 3 convolutions with 64 kernels before being concatenated. Finally, the concatenated feature maps are run through a block of 3 $\times$ 3 convolutions with 320 kernels, batch normalization, and ReLu, to obtain the output of that decoder stage. This way, feature maps at every decoder stage have a depth of 320 and contain information on every scale.

## Experimental settings

All experiments were performed on an Ubuntu 20.04 server, using an AMD Ryzen 9 3900X 12-Core CPU with 64GB system memory and a single NVIDIA Titan RTX 1330 24GB GPU. The software was implemented in python 3.7 using PyTorch 1.5.0 and is based on the work of Cao^47^. It has been extensively modified to address the problem at hand.

### Model selection

In model selection, we perform hyper-parameter tuning (HPT) to increase model performance and reduce training time. The objective is to adapt to the model to achieve the ideal balance between over- and underfitting. To save training time, we will use a single training/validation split for model selection. We monitor loss and Dice scores. We will do that for our method and for all the other considered techniques taken into account. The hyper-parameters can generally be divided into two groups, the model design, and the optimizer hyper-parameters.

On the one hand, the model design hyper-parameters define the model’s capacity in terms of depth (number of recurrent blocks or layers) and width (number of filters), the loss function, dropout rate, and the optimizer. Concerning the model capacity, we will keep the depth and width equal to the default values^20^ while maintaining an even model capacity across the architectures throughout the experimental phase. The dropout rate is the primary source of regularization on the model design side. We keep this value to the default and focus on the optimizer hyper-parameter to adjust regularization strength. Concerning the loss function, we use a combination of Cross-Entropy and Dice^48^ during model selection and comparison, as commonly seen in medical image segmentation, with $\lambda _1 = \lambda _2 = 0.5$.

$$\begin{aligned} loss = \lambda _1\cdot loss_{CE} + \lambda _2\cdot loss_{Dice} \end{aligned}$$

We choose stochastic gradient descent (SGD)^49^ as the optimizer. In an empirical comparison of common types of optimizers, Choi and coauthors^50^ found similar performance between SGD and other optimizers (after learning rate tuning) and a less severe increase of training time with increased batch size.

Optimizer’s hyper-parameters primarily consist of batch size, learning rate (LR), momentum, weight decay (WD), and the number of training epochs. Especially the first four parameters have a significant impact on generalizability and are strongly interconnected^51^. In this work, we consider a maximum number of epochs equal to 50 and an early stopping criterion to stop the training phase if the loss on the validation set does not improve for six consecutive epochs. Focusing on the optimizer’s hyper-parameters, to find suitable values within the hyper-parameter space, we perform a 3-stage manual search individually for each method. We adopt the tuning strategy described in^51^ and choose a batch size as large as possible, only restricted by the GPU. In stage 1, we identify the best learning rate range for the cyclic LR-scheduler while keeping the values for momentum and WD constant. We try different LR ranges within the boundaries of $[5e{-5}, 1.5]$ where each LR$_{base}$ is a tenth of the corresponding LR$_{max}$. In stage 2, based on the best values for the learning rate range for the respective method, we evaluate the best value for the momentum range. Finally, stage 3 builds on the best values for the learning rate and momentum ranges. The values of the hyper-parameters resulting from the HPT phase are reported in Table 2.

### Model comparison

In model comparison, we compare different types of architectures, all of which were previously fine-tuned in the HPT phase, on a set of test images (that were not considered during the model selection stage). To calculate the test metrics, we take the model with the smallest validation loss for each model and fold. To obtain statistically significant results, we compare the test metrics over a 5-fold cross-validation.

### Dataset splitting

We split the dataset at the patient level, and B-scans of a particular patient can not simultaneously occur in the training and test datasets. Two B-scans of a particular patient taken at different points in time but at the same position of the retina are expected to show a high structural correlation. As a consequence, allowing them to occur in both train and test sets would have resulted in information leakage. Furthermore, we stratified the splits according to groups of classes. Considering the three classes IRF, SRF, and PED, this results in seven groups: IRF, PED, SRF, IRF+PED, IRF+SRF, PED+SRF, IRF+PED+SRF. The stratified split guarantees a similar distribution of those groups to generate homogeneity across the datasets and reduce selection bias. For both model selection and model comparison, we relied on nested k-fold cross-validation (CV), as proposed by Raschka^52^. In this work, we apply a slight variation to this approach. We maintain the outer 5-fold CV but we replaced the inner CV with a single split. In this way, we reduce the overall training time, at the cost of losing the possibility of training on every data point at least once.

## Results

### Model selection

In the model selection, we perform hyper-parameter tuning for each of the methods under analysis. To align the models’ capacity in terms of their total number of parameters, we consider two different settings for the Swin-UNETR. First, a version with the default settings as proposed by the original authors, with a feature size of 24, dubbed Swin-UNETR-24. With only 7 Mio. parameters, this version has a far smaller capacity than U-Net (24 Mio.) and U-Net3+ (27 Mio.). We propose a second version of the Swin-UNETR, dubbed Swin-UNETR-48, with a feature size set to 48, which increases the model’s capacity to 27 Mio. parameters, making it comparable to the CNN-based methods.

The results are shown in Table 2. In general, the choice of LR is more relevant for U-Net3+ than for Swin-UNETR. In return, Swin-UNETR is much more sensitive to the choice of momentum and weight decay. In terms of the LR scheduler, a constant LR of $5e{-3.5}$ achieves lower Dice scores for all but Swin-UNETR-48 in comparison to the cyclic LR that oscillates around the same LR value of $5e{-3.5}$. As far as the LR range (a), the U-Net performed best at a range of $5e{-2}$ to $5e{-1}$ and the remaining methods at a step to the power of ten smaller. In terms of momentum, we choose a rather small range of [0.8, 0.95] for U-Net3+ and Swin-UNETR-24 and a high range of [0.85, 0.99] for U-Net and Swin-UNETR-48. For the weight decay, we choose a slightly lower value of $1e{-5}$ for Swin-UNETR-48 while the remaining three methods all work best at a medium value of $1e{-4}$.

**Table 2:** Manual search results of optimizer hyper-parameter tuning, performed in 3 consecutive stages.

LR$_{base}$	LR$_{max}$	U-Net	U-Net3+	Swin-UNETR-24	Swin-UNETR-48
**LR range:** **(a) Mean Dice scores for different learning rate ranges and for the four considered methods. LR**$_{\textbf{base}}$ **and** LR$_{max}$ **represent the lower and upper boundary of the learning rate range respectively. In the case where there is not LR**$_{\textbf{max}}$ **provided, we use a constant LR. In any other case, we use a cyclic LR scheduler. For all methods, the momentum is set to 0.97 and weight decay is set to** $\textbf{1e}{-\textbf{4}}$**. Concerning Swin-UNETR-24, we selected the configuration with** $\textbf{LR}_{\textbf{max}}$ **=** $\textbf{5e}{-2}$ **despite the slightly worse mean Dice score over the next bigger LR range by 1%. The main reason is the strongly increased runtime by a factor of 2 at a small improvement of the validation loss by 0.001 points**					
$5e{-5}$	$5e{-4}$	0.389	–	0.370	0.393
$5e{-4}$	$5e{-3}$	0.438	0.478	0.449	0.452
$5e{-3.5}$	–	0.421	0.444	0.423	0.468
$5e{-3}$	$5e{-2}$	0.432	**0.537**	**0.454**	**0.485**
$5e{-2}$	$5e{-1}$	**0.505**	0.362	0.463	0.459
$5e{-1}$	1.5	0.359	0.169	0.403	0.402

M$_{base}$	M$_{max}$	U-Net	U-Net3+	Swin-UNETR-24	Swin-UNETR-48
**(b) Momentum range: Mean Dice scores for different momentum ranges and for the four considered methods. M**$_{\textbf{base}}$ **and M**$_{\textbf{max}}$ **represent the lower and upper boundary of the momentum range respectively. For the LR**$_{\textbf{base}}$ **and LR**$_{\textbf{max}}$**, we consider values that yielded the best results in the previous stage (displayed in bold). For all methods, the weight decay is set to** $\textbf{1e}{-\textbf{4}}$					
0.80	0.90	0.504	0.536	0.476	0.489
0.80	0.95	0.480	**0.539**	**0.496**	0.461
0.85	0.97	0.505	0.537	0.454	0.485
0.85	0.99	**0.513**	0.524	0.460	**0.497**

Weight decay	U-Net	U-Net3+	Swin-UNETR-24	Swin-UNETR-48	
**(c) Weight decay: Mean Dice scores for different weight decay coefficients and for the four considered methods. For all methods, we consider the values for the learning rate and momentum ranges that yielded the best results in the previous stages**					
$1e{-6}$	0.474	0.520	0.448	0.481	
$1e{-5}$	0.509	0.534	0.451	**0.499**	
$1e{-4}$	**0.513**	**0.539**	**0.496**	0.497	
$1e{-3}$	0.360	0.531	0.477	0.470	
$1e{-2}$	0	0.463	0.435	0.356	

In stage 1 (Tab. a), we find the best values for the learning rate range, in stage 2 (Tab. b) for the momentum range and in stage 3 (Tab. c) for weight decay. The tables show the weighted mean Dice score over the classes of the training epoch calculated on the validation set with the lowest validation loss for the considered hyper-parameter configurations. The best configuration for each method is displayed in bold.

### Model comparison

#### Quantitative

#### Overall performance

The results of the model comparison are summarized in Table 3. Across all classes, U-Net3+ has the highest Dice scores. In terms of mean Dice, it outperforms the other methods by 0.05, while the remaining performers lie within a range of 0.01. Considering the different classes, PED clearly shows the best overall Dice scores followed by SRF when segmented by CNN-based methods or IRF in the case of Swin-Transformer. The latter, however, only shows a weak variance between IRF and SRF of less than 0.01. The two versions of the Swin-UNETR both achieve similar results.

**Figure 1:**
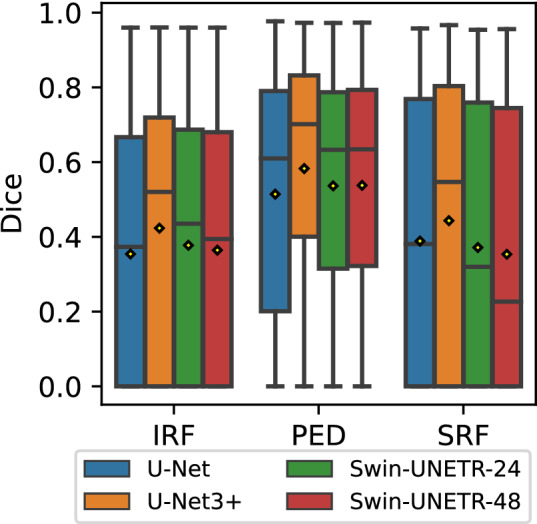
Boxplots of model comparison results per class for Dice score across the entire dataset. The boxes show the middle 50% of the values. Whiskers reach at most 1.5 times the interquartile range (= box height) on top of either end of the box. Points outside the whiskers are considered outliers and are not shown here. The horizontal bar within the box shows the median. The mean is depicted with a yellow diamond.

Looking at the value distribution of the Dice scores depicted in Fig. 1, the general tendencies described in the previous paragraph can be confirmed. However, we can observe a significant variance in the scores across the cases of the dataset. This is especially true for the Dice score of IRF and SRF.

**Table 3:** Method comparison results.

Method	IRF	PED	SRF	MEAN
U-Net	0.354 (0.023)	0.515 (0.027)	*0.391* (0.042)	0.443 (0.029)
U-Net3+	**0.423** (0.028)	**0.582** (0.023)	**0.444** (0.030)	**0.508** (0.026)
Swin- UNETR- 24	*0.379* (0.036)	0.537 (0.013)	0.372 (0.028)	*0.457* (0.023)
Swin- UNETR- 48	0.365 (0.031)	*0.538* (0.014)	0.359 (0.043)	0.451 (0.025)

Mean and (standard deviation (std)) of the Dice scores for the four considered methods calculated on the test set over a 5-fold cross-validation (CV). Results reported per class (columns 2-4) and as a weighted average (column 5 (MEAN)). The best performer is displayed in bold, followed by the second best in italic.

#### Over- and under-segmentation: sensitivity and specificity

To better understand the methods’ tendency to over- or under-segment a class, we report sensitivity and specificity in Table 4. Sensitivity measures the rate of correctly predicted foreground classes, while specificity measures the same quantity for the background class. Reduced sensitivity is due to an increase in false negatives (FNs), meaning the algorithm fails to predict the specific class and suggests the pixel belongs to the background or to an incorrect foreground class. Analog, a reduced specificity is caused by an increase in false positives (FPs). In other words, pixels that belong to the background or an alternate class were falsely predicted to belong to one of the specific foreground classes. Consequently, poor sensitivity represents under-segmentation and poor specificity over-segmentation. Overall, all methods show a clear tendency towards a higher specificity than sensitivity. However, there exist some differences across the classes as well as the methods. The sensitivity values across classes and methods vastly align with the observations reported for Dice. PED achieves the highest values, followed by SRF and IRF across all methods. U-Net3+ shows the highest values across all classes, followed by the Swin-UNETRs, while U-Net clearly shows the poorest performance. Across all methods, PED is the only class achieving a specificity of lower than 0.999. U-Net3+ has the highest value of 0.995, while the values of the Swin-UNETRs are the lowest, with a difference of 0.002.

**Table 4:** Method comparison results.

Method	Sensitivity				Specificity			
	IRF	PED	SRF	MEAN	IRF	PED	SRF	MEAN
U-Net	0.389 (0.078)	0.553 (0.080)	0.534 (0.093)	0.504 (0.082)	0.999 (0.001)	0.994 (0.002)	0.999 (0.001)	0.997 (0.001)
U-Net3+	0.526 (0.047)	0.640 (0.034)	0.600 (0.014)	0.600 (0.033)	0.999 (0.000)	0.995 (0.001)	0.999 (0.000)	0.997 (0.001)
Swin- UNETR- 24	0.497 (0.033)	0.616 (0.061)	0.589 (0.035)	0.578 (0.048)	0.999 (0.000)	0.993 (0.003)	0.999 (0.001)	0.996 (0.002)
Swin- UNETR- 48	0.504 (0.039)	0.628 (0.028)	0.587 (0.045)	0.585 (0.035)	0.999 (0.000)	0.993 (0.002)	0.999 (0.001)	0.996 (0.001)

Mean and (std) of the sensitivity and specificity calculated on the test set over a 5-fold CV per class and as a weighted average.

#### Impact of lesion size

**Figure 2:**
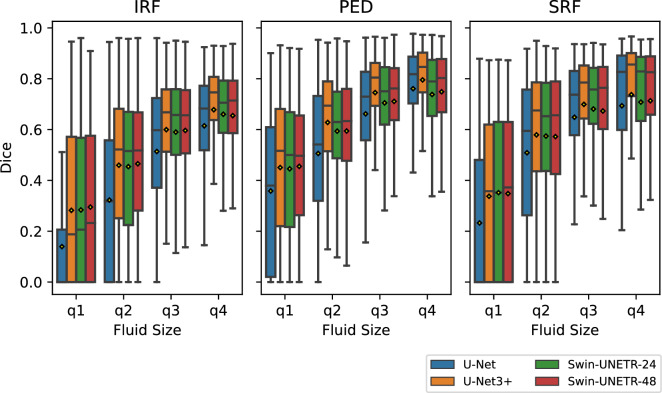
Model comparison results per class and lesion size. The boxplots show the value distribution of the entire dataset for the Dice scores. Lesion sizes are discretized into quartiles from small (q1) to large (q4). The boxes show the middle 50% of the values. Whiskers reach at most 1.5 times the interquartile range (= box height) on top of either end of the box. Points outside the whiskers are considered outliers and are not shown here. The horizontal bar within the box shows the median. The mean is depicted with a yellow diamond.

Figure 2 shows the segmentation performance of the evaluated methods regarding different lesion sizes. The sizes were approximated by the number of pixels per image and class. Generally, performance increases with the lesion size while the variance decreases. Across all lesion sizes, U-Net performs noticeably weaker than the other methods, and U-Net3+ is superior, especially for the PED class. The advantage of U-Net3+ over both Swin-UNETR methods becomes particularly clear for the larger lesion sizes. Within the two Swin-UNETR methods, there exist only slight differences in mean performance. However, a reduced performance variance can be observed for the larger model.

#### Methods computational efficiency

**Figure 3:**
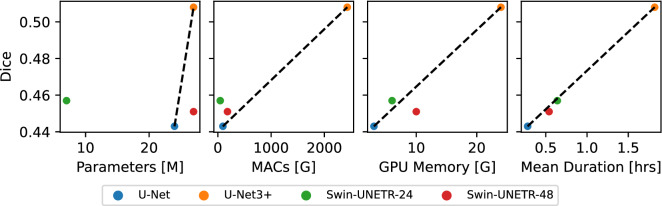
Comparison of the methods’ computational efficiency across four different metrics. The mean cv5-Dice score over the classes is plotted against the total number of trainable parameters (top-left), the number of multiply-accumulate operations (MACs) (top-right), the measured GPU memory used during training (bottom-left), and the mean training duration over the folds (bottom-right). The dotted line represents the relationship between the two methods with the lowest and highest Dice scores.

Figure 3 compares the methods’ performance against their computational efficiency measured by four different metrics. The leftmost two metrics, namely the total number of trainable parameters ($n\_params$) and the number of multiply-accumulate operations (MACs), are both characteristics of a particular architecture, while the rightmost two metrics are measured during the actual training.

In terms of $n\_params$, Swin-UNETR-24 uses far fewer parameters than the remaining three methods. While U-Net and Swin-UNETR-48 have a lower Dice score, U-Net3+ outperforms the remaining methods. As a result, Swin-UNETR-24 and U-Net3+ dominate the remaining two methods in terms of $n\_params$.

MACs represent the linear transformation operations of a neural network, including matrix multiplications and convolutions. Unlike non-linear functions, such as rectified linear units (ReLU) and pooling operations, their computational costs grow quadratically with the network size, making them the main hardware bottleneck.^53^ Here, the value for U-Net3+ is about 25 times higher than the remaining competitors while also showing an increased Dice score. This hugely increased value can be explained by the expensive skip connections of U-Net3+ where at each decoder level one convolution per encoder level plus one extra convolution are added to the network, compared to simple concatenate operations in the U-Net. Amongst the remaining methods, Swin-UNETR-24 has the highest Dice score, again leading to the domination of U-Net3+ and Swin-UNETR-48.

For memory usage, please recall that we used an equal batch size of 16 across the methods. Consequently, the observed differences are fully due to the architecture design and implementation. The dotted line connects the U-Net as the method with the lowest to U-Net3+ with the highest memory usage. Swin-UNETR-24 lies slightly above and Swin-UNETR-48 slightly below that line. We argue that this result corresponds to the slightly more efficient memory usage of Swin-UNETR-24 compared to the remaining competitors.

Last, in terms of training time, three methods roughly lie on the same dotted line. Only the Swin-UNETR-48 lies slightly below that line, reflecting a negligible disadvantage in training time efficiency.

#### Qualitative

**Figure 4:**
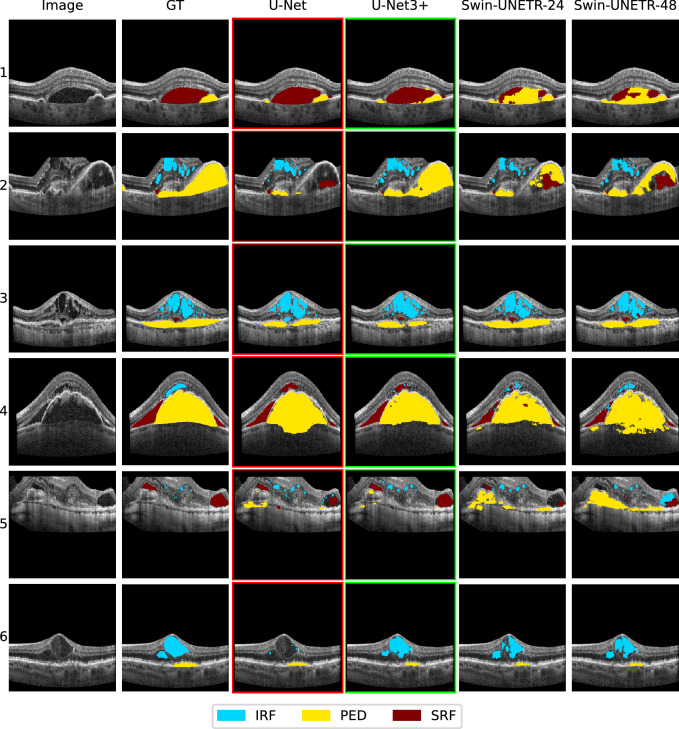
Qualitative model comparison. The columns represent the original image, the reference (GT), and the predictions of the four considered methods from left to right. Each row represents an example case that was selected from the cases with the biggest variance of the mean Dice scores over the folds amongst the four methods. The best and the worst method are highlighted in green and red respectively.

Figure 4 shows typical example cases of misclassifications and differences between the methods. For instance, we can observe that U-Net confuses IRF (light blue) either with the background (row 6) or with SRF (dark red) (row 4), while U-Net3+ performs best in all cases. Concerning the PED (yellow) class, we can observe misclassifications with the background for all methods but U-Net3+ (row 2). Furthermore, ViTs show signs of over-segmentation of PED (rows 4 and 5). SRF gets confused with IRF by Swin-UNETR-48 (row 5). Row 3 shows a case with the presence of all three classes but with a very small SRF area compared to IRF and PED. Here, the U-Net fails to separate the SRF from the IRF areas above, while the other methods perform equally well in detecting the SRF area. However, Swin-UNETR-24 shows advantages for PED and IRF, whereas U-Net3+ tends to over-segment the IRF and under-segment the PED class. For the case shown in row 5, U-Net3+ performs well in detecting the SRF areas while U-Net performs slightly worse, failing to detect the entire area and partially mistaking SRF for IRF. Here, both ViTs perform drastically worse, with Swin-UNETR-24 hardly managing to distinguish the area from the background and Swin-UNETR-48 mistaking large parts with IRF. Furthermore, ViTs confuse SRF with PED (row 1), where the SRF area is uncharacteristically large compared to the PED area. Finally, there exist cases with holes in the segmented areas of IRF (row 6), SRF (row 5), and PED (rows 2 and 4), where U-Net3+ is least affected.

## Discussion

### Model comparison

#### Overall performance

In absolute performance, Swin-UNETR is inferior to the advanced CNN-based architecture of the U-Net3+ in the segmentation of retinal lesions on our dataset. We argue that the complex skip connections in U-Net3+ successfully compensate for the smaller initial receptive field of the CNN encoder, thus resulting in higher segmentation performance. This is supported by the observed performance advantages of Swin-UNETR over the less advanced CNN-based architecture of the U-Net.

However, it is worth mentioning that the version of Swin-UNETR used in this work is far less computationally expensive than U-Net3+. This motivates further investigations into the network’s architecture to increase Swin-UNETR’s model capacity and the resulting impact on segmentation performance. In this work, we investigated the increase in the feature size, which did not yield any relevant performance improvement. Possible further options include the increase of the model depth, patch resolution, or the number of attention heads that can be explored in future work.

Furthermore, a known limitation of ViTs is their need for large amounts of training data. Even though this caveat is already addressed by the hybrid structure of the Swin-UNETR, we suggest further investigations of the effect of differently sized training data, data augmentation, or the use of pre-training on medical image data from other domains.

#### Class-wise performance

In terms of Dice score, all methods performed best in the PED class followed by either SRF (CNNs) or IRF (Swin-UNETRs). The differences between the classes are bigger for the Swin-UNETRs than for U-Net and U-Net3+, with values for the std across the classes ranging from 0.084 for the U-Net and 0.086 for the U-Net3+ to 0.093 and 0.010 for Swin-UNETR-24 and Swin-UNETR-48 respectively. In the related work, the order between IRF, SRF, and PED varies for each method also when tested on the same dataset. This lets us assume that it depends not only on the dataset used to obtain the score but also on the segmentation method itself. The differences between the classes tend to be smaller than observed in this work. Contrary to the order, however, we can observe a dependency solely due to the characteristics of the dataset. The related works that used the publicly available RETOUCH^54^ test dataset all report relatively small differences in Dice score between the classes with a std of 0.020 to 0.025^55–58^ while the ones using private datasets report larger values of 0.075^59^ and 0.087^60^. Consequently, we assign the reason for the spread of the Dice scores between the classes primarily to the characteristic of the dataset. To achieve more general results, future work can repeat our experiments on publicly available datasets.

Furthermore, we can observe a strong imbalance between sensitivity and specificity. While the sensitivity ranges between 0.389 and 0.640 depending on the class and method, the specificity mostly reaches a value of 0.999. We argue that the overall very high specificity and resulting rare occurrence of over-segmentation is a response to the highly unbalanced dataset with a strongly overrepresented background class with an average area coverage of nearly 97%. Most signs of over-segmentation can be observed in the PED class, which can be measured by a slightly reduced specificity. This can be explained by PED being the most represented foreground class. On average, PED covers 2.66% of the image area while IRF and SRF only cover 0.95% and 1.23% respectively. Swin-UNETRs show the tendency of PED over-segmentation slightly stronger than U-Net3+. Examples of this behavior can be observed in Fig. 4 rows 4 and 5.

#### Impact of lesion size

Across all methods, we observe an increase in the Dice score with the lesion size. This confirms the findings of previous related work^59^. The performance advantage of U-Net3+ becomes obvious with larger lesion sizes. This is particularly true for the PED class, which tends to cover larger areas in the image than the two remaining classes. It is somewhat counterintuitive to the theoretically high robustness of ViTs to variations in lesion sizes and proves once more the power of U-Net3+’s advanced skip connections. We know, from related work, that multi-scale feature extraction methods like advanced skip connections can improve the detection of lesions of different sizes. However, we expected the dynamically sized receptive field of ViTs’ attention heads to be even more potent. Possibly, the local attention mechanism of the Swin Transformer backbone used in Swin-UNETR hinders that advantage. We recommend future work to experiment with different ViT backbones that use global instead of local attention heads. A further limitation of our approach is the proxy used to measure the lesion size. We used a simple pixel count approach that is unaware of multiple instances of one class within a particular image. As we reached the same overall results as related work, we do not expect fundamentally different findings with an improved proxy. Still, future work can reproduce the experiments with more precise proxies that are agnostic to class instances.

### Further limitations

Limitations of this work are mainly related to the private dataset used here.

In this work, we consider every 2d image separately, without including their relationship in the 3-dimensional space. This is due to the limited capacity of the reading center, where the manual labeling of the reference is only performed on five, non-consecutive slices per volume. This makes embedding them into a 3d context impossible consequently preventing the precise quantification of the lesion volume. Coarsely sampled OCT volumes can even miss certain lesion instances entirely^61^. Also, related work shows possible improvements in segmentation performance when adding 3d context, which we can not reproduce based on the dataset used in this work^62^.

Furthermore, our data is obtained from devices of a single vendor only. Related work has shown strong dependencies of segmentation performance across image data from different vendors^56–58,62^. In future work, we will consider image data from different vendors to compare the cross-device generalizability capabilities of ViTs to CNN-based methods.

## Conclusion

Regarding the key contributions of this work, we conclude the following. **C.1**—In adapting Swin-UNETR to the domain of automatic retinal OCT lesion segmentation, this work contributed to a recent research strand that focuses on the use of ViTs for the automated segmentation of retinal lesions in OCT images. In particular, in our study, we used a hybrid Transformer-CNN that, unlike the pure transformer used in recent contributions^25,26^, is less training data demanding and thus does not require pretraining. In this work, the adaptation of the Swin-UNETR is limited to experimenting with different feature sizes. We demonstrated that increasing feature size does not yield any performance increase. As the next steps, we suggest investigating the effect of the model depth, patch resolution, or the number of attention heads. **C.2**—Even though we were not able to demonstrate the superiority of ViTs over CNN-based approaches in terms of absolute performance, the demonstrated advantages in computational efficiency motivate future work to investigate changes in the model’s architecture for further performance improvements. **C.3**—As far as analyzing the effect of lesion size on the segmentation performance, we can confirm the overall findings of related work of increasing performance with the lesion size. Comparing the segmentation performance of ViTs with CNN-based approaches we found that Swin-UNETR shows room for improvement particularly with larger lesions.

In the clinical context of retinal lesion segmentation of OCT images, this work is a valuable contribution for improving current DL-based segmentation algorithms. Possible applications include the support of practitioners with segmentation suggestions made by the algorithm and the use of the segmentation results as a source of input for subsequent prediction questions like the treatment intensity. At this point, a full replacement of the practitioner in identifying the lesions is not recommendable until a further reduction of the segmentation error is reached. Considering the shown imbalance between positive and negative prediction errors, it is up to the specific field of application or to the individual practitioner even, to decide. For the decision-making support in the clinic, on the one hand, we argue that the here observed imbalance towards the specificity is generally not favorable. The system should be more balanced or even lean towards an over-segmentation to raise the practitioner’s attention towards a certain anomaly, that the human can then reject if false. The greatest danger is failing to detect early signs of nAMD resulting in a delayed initialization of the treatment, which can dramatically worsen the visual outcome. As input to subsequent prediction algorithms, on the other hand, we suggest identifying the balance between over- and under-segmentation that leads to the best overall Dice score. Finally, to foster the use of automated segmentation systems, it would be essential to assess whether an automated suggestion can speed up the diagnostic process and lead to more uniform results across the practitioners.

## Data Availability

The data that support the findings of this study were not publicly available. Data are, however, available from Kai Rothaus upon reasonable request and after the permission of the St. Franziskus Eye-Center in Muenster.
